# Cognitive Impairment in MRI-Negative Epilepsy: Relationship between Neurophysiological and Neuropsychological Measures

**DOI:** 10.3390/diagnostics13182875

**Published:** 2023-09-07

**Authors:** Vasileios Papaliagkas, Chrysanthi Lokantidou-Argyraki, Panayiotis Patrikelis, Georgia Zafeiridou, Martha Spilioti, Theodora Afrantou, Mary H. Kosmidis, Marianthi Arnaoutoglou, Vasileios K. Kimiskidis

**Affiliations:** 1 Department of Biomedical Sciences, School of Health Sciences, International Hellenic University, 57001 Thessaloniki, Greece; 2 Laboratory of Cognitive Neuroscience, School of Psychology, Aristotle University of Thessaloniki, 54124 Thessaloniki, Greece; 3 First Department of Neurology, Medical School, Aristotle University of Thessaloniki, 54124 Thessaloniki, Greece; 4 Second Department of Neurology, Medical School, Aristotle University of Thessaloniki, 54124 Thessaloniki, Greece; 5 Laboratory of Clinical Neurophysiology, Aristotle University of Thessaloniki, 54124 Thessaloniki, Greece

**Keywords:** event-related potentials, epilepsy, P300, anti-seizure medication

## Abstract

Background: Epileptic patients frequently encounter cognitive impairment. Functions that are mostly affected involve memory, attention, and executive function; however, this is mainly dependent on the location of the epileptic activity. The aim of the present study is to assess cognitive functions in MRI-negative epilepsy patients by means of neurophysiological and neuropsychological measures, as well as study the concept of transient cognitive impairment in patients with epileptiform discharges during EEG acquisition. Methods: The patients were enrolled from an outpatient Epilepsy/Clinical Neurophysiology clinic over a time period of 6 months. The study sample comprised 20 MRI-negative epilepsy patients (mean age ± standard deviation (SD), 30.3 ± 12.56 years; age range, 16–60 years; average disease duration, 13.95 years) and 10 age-matched controls (mean age ± SD, 24.22 ± 15.39 years), who were also education-matched (*p* > 0.05). Patients with epileptogenic lesions were excluded from the study. Informed consent was obtained from all subjects involved in the study. Auditory ERPs and the cognitive screening tool EpiTrack were administered to all subjects. Results: Latencies of P300 and slow waves were prolonged in patients compared to controls (*p* < 0.05). The ASM load and patients’ performance in the EpiTrack maze subtest were the most significant predictors of P300 latency. A decline in the memory, attention, and speed of information processing was observed in patients with cryptogenic epilepsy compared to age-matched controls, as reflected by P300 latency and EpiTrack scores.

## 1. Introduction

Cognitive deficits and behavioral sequelae are frequently encountered in patients with epilepsy (in approximately 70% of chronic epilepsy cases). The deleterious effects of epilepsy in patients’ neurobehavioral function can be determined with a variety of causes such as structural brain lesions, seizures, epileptic dysfunction, and medical treatment [1].

Besides the aforementioned chronic and progressive effects of epilepsy on cognition, transient cognitive impairment (TCI), first introduced by Aarts et al. [2], is a transient state of aberrant cognitive function associated with subclinical interictal epileptiform discharges. In this study, 50% of the patients showed transient cognitive impairment that was associated with interictal epileptiform discharges (ED). TCI caused by these epileptiform discharges followed a lateralized pattern, i.e., ED starting in the left hemisphere caused errors in verbal tasks, while those generated in the right hemisphere caused impairments in non-verbal tests. It appears that ED cause a temporal disruption of the function of the brain region involved in the generation of the epileptiform activity as well as nearby interconnected areas. ED have often been associated with memory impairment, executive dysfunction, as well as behavioral disturbances, in adults and children with epilepsy. The prevalence of TCI is believed to be low, occurring in approximately 2.2% of patients referred for a routine electroencephalogram (EEG). However, others have reported a TCI prevalence of 33% [3]. 

Studies specifically addressing neuropsychological function in cryptogenic epilepsy are relatively scarce [4]. Cognition is more likely impaired in patients suffering seizures of a symptomatic than idiopathic and cryptogenic etiology [5,6]. Yet attention and memory problems have been estimated in about 30% of newly diagnosed patients with single or several seizures of a cryptogenic origin [7]. The most important factor in shaping cognitive performance in cryptogenic epilepsy appears to be lower educational attainment, a fact consistent with the cognitive reserve theory in epilepsy [8], while the number of seizure types, epilepsy duration, and polytherapy also appear to be relevant factors [9]. An increased number of seizure types leads to the disturbance of more complex brain circuits (e.g., hippocampal circuits), determining neuropsychological impairment in cryptogenic epilepsies [10]. Neuronal loss is also proportional to the duration of epilepsy [11], while adequate treatment can improve cognitive function in previously cognitively impaired patients [9]. The lack of morphological abnormalities is likely to render seizure localization and lateralization more challenging in cryptogenic than in lesional epilepsy. In such cases, neuropsychological assessment may be of help in the detection of the epileptogenic zone. As these patients are also at an increased risk for postoperative cognitive decline when neurosurgery is considered to control their seizures [9], identifying potential cognitive decline is of utmost importance.

TCI has also been studied in animal models, although studies observing the acute effect of ED on cognitive functions are fewer compared to human studies. Petrucco et al. [12] studied epileptic activity in the visual cortex of mouse models and observed that cortical processing can be impaired in areas that are far from the brain region responsible for the generation of ED. The results of this study agree with several EEG and fMRI studies in humans that observed changes of the BOLD signal in brain structures far from the epileptic generator [13]. Therefore, it appears that the propagation of ED to connected brain regions may also contribute to TCI [14].

A representative example of how chronic epileptiform activities cause neurodevelopmental deficits is observed in epileptic encephalopathies such as Landau–Kleffner and West syndromes. On the other hand, in epileptic syndromes like Dravet syndrome, cognitive impairment is not only associated with EEG abnormalities but also with the underlying genetic pathology of the syndrome [15].

**Figure 1:**
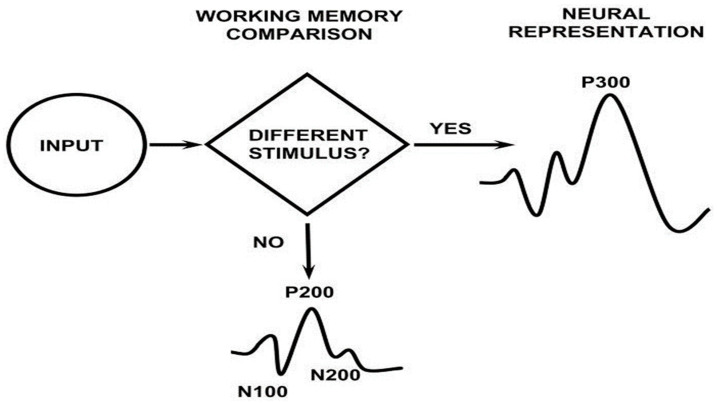
Context updating theory of P300 (Adopted by Polich [18]).

Event-related potentials (ERPs) reflect the summed activity of postsynaptic potentials and are generated when millions of similarly oriented pyramidal neurons depolarize synchronously in response to an infrequent or so-called “target” stimulus [16]. They have been frequently used as an accurate neurophysiological marker of cognitive function together with neuropsychometric and neuroimaging tests. The P300 component is a positive deflection that occurs at a latency of about 300 ms after stimulus onset. It is elicited with the selection of a specific processing information strategy, while it depends on the way a subject tends to respond to future stimuli; it reflects the expectancies’ formation process, strategic solutions, and memory processing [17]. The P300 amplitude represents cognitive events underlying “tasks that are required in the maintenance of working memory” [18], since it is an ERP component that is thought to reflect updating operations of the stimulus context [19,20]. P300 latency has been linked to the speed of stimulus classification during memory updating [21]. In healthy subjects, P300 latency negatively correlates with the timing of attentional allocation processes that varies across individuals [22]. In general, two P300 components have been described [14]. The earlier frontocentral P3a component occurs when the subject is presented with an unexpected stimulus in the absence of any prior instruction and the temporoparietal P3b component reflects an update in working memory processes following the presentation of new information according to the context updating theory (Figure 1).

The P300 component has been studied previously as a marker of cognitive dysfunction in numerous neurological diseases, including epilepsy, since it reflects attention and memory processing [6,10]. Studies of P300′s characteristics in epileptic patients have mainly observed increased P300 latencies and lower P300 amplitudes compared to normal subjects. Prolonged P300 latency has been linked to a reduced speed of information processing [18]. The present study aimed to evaluate cognitive processing in patients suffering from cryptogenic epilepsy by means of neurophysiological and neuropsychological measures and seek possible relationships between measures of cognitive performance and clinical characteristics of the disease. Moreover, the authors studied the concept of TCI in a patient subgroup with epileptiform discharges (ED) during ERP acquisition, by assessing the ED influence on ERP component characteristics.

## 2. Materials and Methods

This study was conducted at the Laboratory of Clinical Neurophysiology in AHEPA University Hospital of Thessaloniki after approval by the research ethics committee of International Hellenic University (ethics committee approval code: 3/29.5.2023). The patients were enrolled from the outpatient Epilepsy/Clinical Neurophysiology clinic over a time period of 6 months. Study participants constituted a sample of convenience, based on the inclusion criteria, and experimental quantitative data were collected retrospectively. Specifically, the study sample comprised 20 adult epilepsy patients, with mainly generalized epilepsy, who were followed regularly in the outpatient clinic with no identifiable etiological lesion detected on MRI imaging (MRI-negative epilepsy) (mean age ± standard deviation (SD), 30.3 ± 12.56 years; age range, 16–60 years; average disease duration, 13.95 years), and 10 age-matched controls (mean age ± SD, 24.22 ± 15.39 years), who were also education-matched (*p* > 0.05). Patients with epileptogenic lesions were excluded from the study. The diagnosis was made by experienced adult neurologists specialized in epilepsy. The participants underwent a thorough neurological examination and structural brain MRI (high-resolution T1, T2, FLAIR, and diffusion-weighted imaging sequences) before inclusion in this study. All participants provided written informed consent. The anti-seizure medication (ASM) load was calculated using the ratio of the prescribed daily dose to the defined daily dose (DDD). DDD is the assumed average daily drug dose defined by WHO. Our patients were all on a polytherapy regimen, mainly with Levetiracetam (LEV) and valproic acid (VAP).

### 2.1. Neuropsychological Testing

EpiTrack, a brief screening measure for detecting cognitive impairment, was administered to all patients by a trained psychologist. This test battery comprises six subtests (Trail-Making Test parts A and B, a test of response inhibition, a backward digit span, written word fluency, and a maze test) covering the domains of processing speed, planning, word fluency, and working memory [23]. It is especially sensitive to cognitive changes associated with antiepileptic medication. The duration of the test was approximately 10–15 min. The maximum score is 49 points and the cutoff for significant impairment is ≤28 points.

### 2.2. Recording Parameters

Auditory ERPs were assessed using a Nihon Kohden Neuropack MEB2300 device. All participants were subjected to the classic oddball paradigm with binaural auditory stimuli at a 70 dB sound pressure level and a 10 ms rise/fall and 100 ms plateau time, presented using earphones. The auditory stimuli were presented in a random sequence with target tones (oddball) of 2000 Hz that occurred 20% of the time and standard tones of 1000 Hz that occurred 80% of the time at a 0.5 Hz rate. All subjects were asked to press a button with their right thumb after every oddball tone was heard, and the reaction time (RT) was subsequently measured.

EEG activity was recorded (filter bandpass, 0.1–50 Hz; analysis time, 1 s) using scalp AgCl electrodes affixed with electrode paste (Elefix Nihon Kohden, EEG paste Z-401 CE, Nihon-Kohden Europe GmbH, Rosbach, Germany) at Fz, Cz, CPz, Pz, T3, and T4 sites according to the 10/20 system referred to as linked earlobe electrodes, with a left-hand ground. All electrode impedances were <5 kΩ [24].

Thirty responses recorded with the target stimuli were averaged, and data from two consecutive trials were obtained. Latencies and amplitudes of N200, P300, and the slow wave (SW), recorded from the Cz site, were considered for this study. In seven patients, epileptiform discharges (ED) occurred during ERP acquisition. ED were defined as spike and/or sharp wave discharges alone or followed by slow waves that clearly stand out from background rhythms. The short-term impact of ED on cognition was tested by calculating ERPs and RTs in two different conditions: with and without ED.

### 2.3. Statistical Analysis

The statistical analysis was performed using SPSS 23.0 for Windows (SPSS, Inc., Chicago, IL, USA). All data were normally distributed (Shapiro–Wilk test). Neuropsychological test scores and ERP parameters of the two groups were compared using an unpaired Student’s *t*-test. The linear relations between the neurophysiological (ERP parameters) and neuropsychological test scores (EpiTrack total score and subtest scores) were studied using Pearson’s correlation r. A multiple regression analysis was performed with P300 wave latency as the dependent variable; *p* < 0.05 was set as the criterion of statistical significance. 

## 3. Results

**Figure 2:**
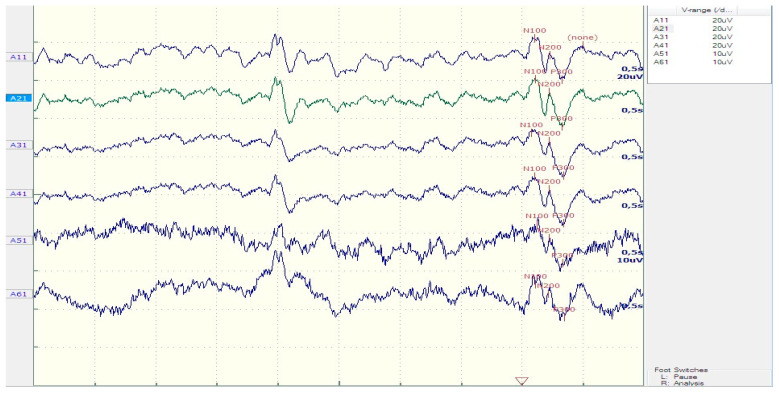
Average of ERP waveforms in epileptic patients recorded with the following electrodes: A11, Fz; A21, Cz; A31, CPz; A41, Pz; A51, T3; and A61, T4. The recording with the Cz electrode is depicted in green, all other recordings are depicted in blue.

Demographic and clinical characteristics of the patients and controls are shown in Table 1. The grand average ERP waveforms of each electrode site are depicted in Figure 2. The mean ± SD P300 latency was 359.73 ± 39.1 ms in the patients and 318.13 ± 23 ms in the controls. P300 and SW latencies were significantly longer in patients than controls (*p* = 0.005 and *p* = 0.05, respectively).

**Figure 3:**
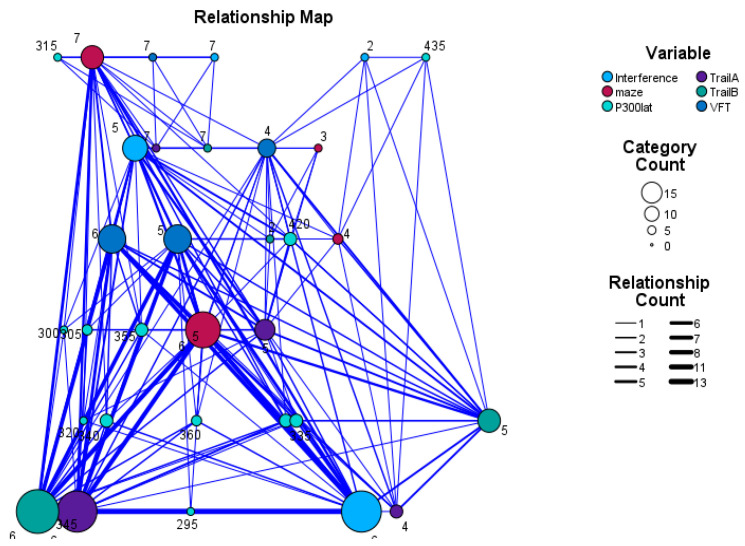
A relationship map that represents the relationship between P300 latency and the performance of the patients in Epitrack subtests. The larger nodes and the thicker lines represent more robust correlations.

The mean amplitude of the P300 component was 16.11 mV in patients and 17.86 mV in controls, with no statistically significant differences (*p* = 0.6). Conversely, a statistically significant difference was evident only in the Trail-Making A and B subtests of EpiTrack, but no other cognitive variables (Table 2). Significant negative correlations were observed between P300 latency and all EpiTrack subtests (*p* < 0.05) (Table 3). Moreover, a relationship map was created between P300 latency and performance of the patients in Epitrack subtests (Figure 3).

In patients who developed ED during EEG recording, the total number of single trials with ED was 300, and that without ED was 683.

No difference was observed between ERP characteristics of N200, P300, and SW components and RT (*p* > 0.05) in single trials with or without ED.

P300 latency and the interference subtest of EpiTrack were significantly correlated with ASM load (*p* = 0.041 and *p* = 0.023, respectively; Table 3). The multiple regression analysis with P300 latency as a dependent variable revealed that the ASM load and patients’ performance in the EpiTrack maze subtest were the most significant predictors of P300 latency (R^2^ = 0.817, Adjusted R2 = 0.634, F = 4.459; *p* < 0.05) (Table 4). No association was found between disease duration and ERP characteristics.

## 4. Discussion

A decline in the memory, attention, and speed of information processing was observed in patients with cryptogenic epilepsy compared to age-matched healthy controls, as reflected by P300 latency and EpiTrack scores. This was mostly due to the ASM load, a finding that agrees with those of previous studies [25,26]. ASM effects are considered to be toxic for cognitive functions given their negative impact on attention, RTs, and the speed of information processing. A few studies have assessed ASM effects on cognition by means of neurophysiological markers such as ERPs. In particular, Chen et al. [27] showed that phenobarbital increased P300 latency, which was unaffected by VAP and carbamazepine. Data from both healthy volunteers and epilepsy patients suggest that LEV exerts few adverse effects on cognition [28,29], while among the older generation, ASMs, VAP, carbamazepine, and phenytoin are less aggressive with respect to cognitive functions with relatively little effects on concentration, memory, information processing speed, or word fluency [30]. Interestingly, evidence regarding the cognitive side effects of ASMs over short periods of up to a year remains inconclusive because of methodological issues. Overall, older ASMs (e.g., phenobarbital) have a more negative impact on cognition than a placebo, a nondrug condition, and newer ASMs, with the exception of topiramate, which has the greatest risk of cognitive impairment irrespective of the comparator group [28].

In our study, a significant correlation between P300 latency and ASM load was observed. Although our results agree with early evidence that suggested a significant correlation between the serum concentration of ASM and P300 [31,32], others [33,34] have failed to replicate these findings. In line with the latter studies, Soysal and associates [35] found a greater prolongation of the P300 latency in cryptogenic focal epilepsy relative to their age-matched control group, pointing to the role of the type of epilepsy rather than that of the type or serum level of ASM.

Evidence [36] on patients with cryptogenic focal epilepsy of a temporal and extra temporal origin points to the influence of epileptic activity on the prefrontal network integrity as a possible underlying problem of memory impairment. The present study showed impaired working memory and encoding, which represent processing milestones for episodic memory. Such findings are related to reduced functional connectivity in the prefrontal areas, rather than to hippocampal pathology as in the case of mesial temporal lobe epilepsy. fMRI studies have also shown a decrease in the clustering coefficient, i.e., the connection probability between adjacent brain regions, as well as an increased path length, i.e., the distance between pairs of nodes in the graph, which was found to be associated with cognitive impairment in patients with cryptogenic epilepsy [37].

Working memory impairment is associated with aberrant prefrontal cortex dynamics [38]. In particular, the frontal prominence of classical generalized 3/s spike-wave absences may be of help in understanding ictal neurobehavioral phenomena arising within the frontal lobes. From a neuropsychological point of view, the distinctive type of disturbed consciousness in classical absences is supposed to rely upon the ictal “arrest of the working memory” [39]. This may also serve to account for working memory impairments in cryptogenic epilepsies, since working memory represents the very early, transient aspects of information processing [40].

Several previous studies have shown significant correlations between P300 latencies and neuropsychological test performance. In particular, an inverse correlation was observed between P300 latency and the total scores on the Wechsler Intelligence Scale for Children-Revised (WISC-R) and those of the Wechsler Memory Scale in pediatric epilepsy [33]. In the present study, P300 latency correlated with all EpiTrack subtests. Our patients’ decreased performance in Trail-Making A and B subtests is suggestive of problems in visual scanning, concentration, and motor speed, as well as dividing and shifting attention (mental flexibility), respectively. ASMs exert their negative influence on cognition by suppressing neuronal excitability or enhancing neuronal inhibition. Attention, vigilance, and psychomotor speed are among the most common domains adversely affected with ASM, a fact also observed in our patients’ Trail-Making A performance. Secondary effects can also manifest on other aspects of cognition, such as problems with executive functions. Impaired Trail-Making B performance may either reflect executive dysfunction and/or the known secondary–systemic effects of arousal deregulation (see below; [41]), and the latter emphasizes the role of aberrant reticulo-thalamo-cortical functional networks in determining, among other clinical factors, cognitive dysfunction. We herewith found that the Porteus Maze test, a measure of executive functioning and visuospatial abilities (planning and foresight), emerged as an important predictor of patients’ overall cognitive impairment, a finding consistent with existing studies, which highlights the presence of executive dysfunction in adolescents with Idiopathic Generalized Epilepsy (IGE) (e.g., [1]). On the other hand, no correlation was observed between N200 latency, which indicates early cognitive elaboration concerning the subject’s attention, and Epitrack subtest performance, suggesting that early preattentive storage is not affected in the early stages of cognitive processing in patients with cryptogenic epilepsy. In addition, we found no association between disease duration and ERP characteristics, in line with previous evidence [12].

Our findings are likely to replicate those of previous research, which revealed an impaired speed of information processing in patients with epilepsy [42,43]. The speed of information processing is a salient area of neuropsychological impairment in patients with MRI-negative epilepsy, potentially reflecting the pathophysiological processes underlying epilepsy and the effects of the ASM load, rather than interictal epileptic activity [21,22]. Recently, it has been suggested that IGE patients’ neuropsychological deficits may represent indirect–secondary manifestations of a primary cortical tone deregulation inherent to IGEs’ pathophysiology. In particular, IGEs’ worse auditory-vigilance and inhibitory-control performance, as compared to its visual counterpart, appears to account for a greater vulnerability of the auditory information processing system to the effects of idiopathic generalized seizures [41]. These findings may apply to our patients’ affected auditory oddball P300 latency and executive and visuospatial dysfunction, since the frontal lobes, especially the right one, are likely to modulate the arousal levels to produce adequate orientation responses towards the task at hand. The prefrontal cortex has rich bilateral connections with the inferior parts of the reticular formation, strongly contributing to cortical tone modulation, which is modified in light of various task demands, and through the mediation of language, i.e., a given “problem’s” formulation induces orienting responses and thus an increase in vigilance [44]. 

We were unable to identify any TCI in those few patients presenting frequent ED, a fact that may be partly due to the small-sized group, as well as the brief duration of the neuropsychological measures implemented, constituting a limitation of this study. Future studies may benefit from the administration of Continuous Performance Tests in which larger time interval transient aberrant neurophysiological phenomena may arise. Consequently, we can only speculate that a direct impact of ED on cognitive functions may not be as prominent in epilepsy patients as mentioned in other studies [2]. Various factors may account for the above finding: First, most ED occurred during non-target stimuli, and only a small percentage of them occurred during target stimuli typically eliciting P300; this is in accordance with evidence [45] showing TCI when ED occurred during a stimulus. Second, the type of ED may also affect the occurrence of TCI. Patients with generalized discharges of >3 s are more likely to develop TCI than those with focal ED or with generalized discharges of <3 s [46]. 

Potential limitations to the generalizability of the findings of the present study should be mentioned. The first is the small sample size; although, cryptogenic epilepsy is difficult to diagnose and distinguish from other types of epilepsy. The second concerns the ERP paradigm that was used to elicit N200, P300, and SW responses, which was a simple oddball paradigm. Possibly, an ERP paradigm, making use of a more demanding task, should be implemented to detect TCI more accurately in patients with an increased frequency of ED. Thus, the conclusions of this study should be considered with caution, since further research with a larger sample size is needed to obtain evidence that is more reliable. Moreover, as observed in previous ERP studies, the P300 component is a reliable and robust electrophysiological marker for objectively assessing cognitive functions in patients with epilepsy, and the combined approach with sensitive neuropsychological screening tools, such as EpiTrack, can provide valuable and accurate data for detecting and evaluating cognitive deficits in patients with epilepsy. Hopefully, future studies including wider neuropsychological batteries assessing multiple cognitive domains, along with their registered ERPs, will address more carefully the issue of cognitive impairment and its neurophysiological correlates in cryptogenic epilepsy.

The results of this study were presented as a conference abstract with the following title: Cognitive impairment in MRI-negative epilepsy: Relationship between neurophysiological and neuropsychological assessments. EPR 2046 EAN Lisbon 2018.

## Figures and Tables

**Table 1:** Demographic and ERP characteristics of patients and controls.

	Patients	Controls	*t*-Test (*p*)
Age (years)	30.3 ± 12.56	24.22 ± 15.39	0.07
N200 latency (ms)	223.75 ± 26.9	212.5 ± 18.3	0.23
P300 latency (ms)	359.73 ± 39.1	318.13 ± 23	0.005 *
SW latency (ms)	492.36 ± 50.72	451.25 ± 41.1	0.05
N200 amplitude (μV)	8.64 ± 3.8	7.83 ± 1.85	0.52
P300 amplitude (μV)	16.12 ± 7.45	17.86 ± 7.02	0.59
Reaction time (ms)	900.25 ± 206.9	833.38 ± 94	0.59

Statistically significant correlations are marked with an asterisk (*).

**Table 2:** Comparison of patients and controls on Epitrack subtest performance.

	Patients	Controls	*p*-Value
Total Score	26.94 ± 3.77	29.87 ± 2.16	0.055
Interference	5.44 ± 1.03	5.75 ± 0.7	0.62
Trail A	5.31 ± 0.79	6 ± 0.53	0.038 *
Trail B	5.31 ± 1.01	6.12 ± 0.35	0.040 *
Maze	5.68 ± 1.19	6.37 ± 0.51	0.137
Verbal fluency test	5.06 ± 0.85	5.62 ± 0.74	0.128
ASM load	2.54 ± 1.70		

Statistically significant differences are marked with an asterisk (*).

**Table 3:** Correlation coefficient r between studied parameters (* statistically significant correlation, *p* < 0.05; ** highly statistically significant correlation, *p* < 0.001).

	N200 Latency	P300 Latency	ASM Load	Trail A	Trail B	Maze	VFT	Interference
N200 latency	1	0.323	0.340	0.033	−0.137	0.066	0.138	−0.085
P300 latency	0.323	1	0.472 *	−0.565 *	−0.564 *	−0.734 **	−0.432	−0.771 **
ASM load	0.340	0.472 *	1	−0.301	−0.436	−0.263	−0.505 *	−0.564 *
Trail A	0.033	−0.565 *	−0.301	1	0.450	0.321	0.166	0.637 **
Trail B	−0.137	−0.564 *	−0.436	0.450	1	0.636 **	0.438	0.307
Maze	0.066	−0.734 **	−0.263	0.321	0.636 **	1	0.478	0.497
VFT	0.138	−0.432	−0.505 *	0.166	0.438	0.478	1	0.421
Interference	−0.085	−0.771 **	−0.564 *	0.637 **	0.307	0.497	0.421	1

ASM: Anti-seizure Medication, Trail A: Trail-Making Test Part A, Trail B: Trail-Making Test Part B, VFT: Verbal Fluency Test.

**Table 4:** Correlations between P300 latency and demographic, clinical, and cognitive variables.

Coefficients			
	Beta	*p*-Value	95% CI for *p*
Age	0.389	0.274	−1.185 3.576
ASM load	0.484	0.053	−0.181 23.291
Trail A	−0.332	0.231	−50.698 14.604
Trail B	0.223	0.401	−14.115 31.292
Maze	−0.674	0.034	−41.228 −2.227
Verbal fluency test	0.245	0.445	−22.763 46.474
Disease duration	−0.308	0.498	−3.905 2.093
Constant		0.004	

## Data Availability

Data sharing is not applicable to this article.
